# Supplementary material for: Amyloid fibril structures link CHCHD10 and CHCHD2 to neurodegeneration
Source: Nat Commun. 2025 Aug 2;16:7121. doi: 10.1038/s41467-025-62149-3 (PMC12318133; doi:10.1038/s41467-025-62149-3)
Supplement: Supplementary file 1 — Supplementary Information [file 41467_2025_62149_MOESM1_ESM.pdf]

Supplementary Information: Amyloid fibril structures link CHCHD10 and CHCHD2 to neurodegeneration.

Guohua Lv[+,1], Nicole M. Sayles[+,2], Yun Huang[+,3,4], Chiara Mancinelli[+,1], Kevin McAvoy[2], Neil A. Shneider[5], Giovanni Manfredi[2], Hibiki Kawamata[*,2], David Eliezer[*,1,2]

[1]Department of Biochemistry, Weill Cornell Medicine, 1300 York Avenue, New York, NY 10021, United States.
[2]Feil Family Brain and Mind Research Institute, Weill Cornell Medicine, 407 E 61st Street, New York, NY 10065, United States.
[3]Department of Physiology & Biophysics, Weill Cornell Medicine, 1300 York Avenue, New York, NY 10021, United States.
[4]Howard Hughes Medical Institute, Chevy Chase, Maryland 20815, United States.
[5]Department of Neurology, Center for Motor Neuron Biology and Disease, Columbia University Irving Medical Center, 630 W 168th Street, New York, NY 10032

[+]These authors contributed equally to this work
[*]Corresponding authors

Contents:

I. **Supplementary Figures 1-20**
II. **Supplementary Tables 1-2**

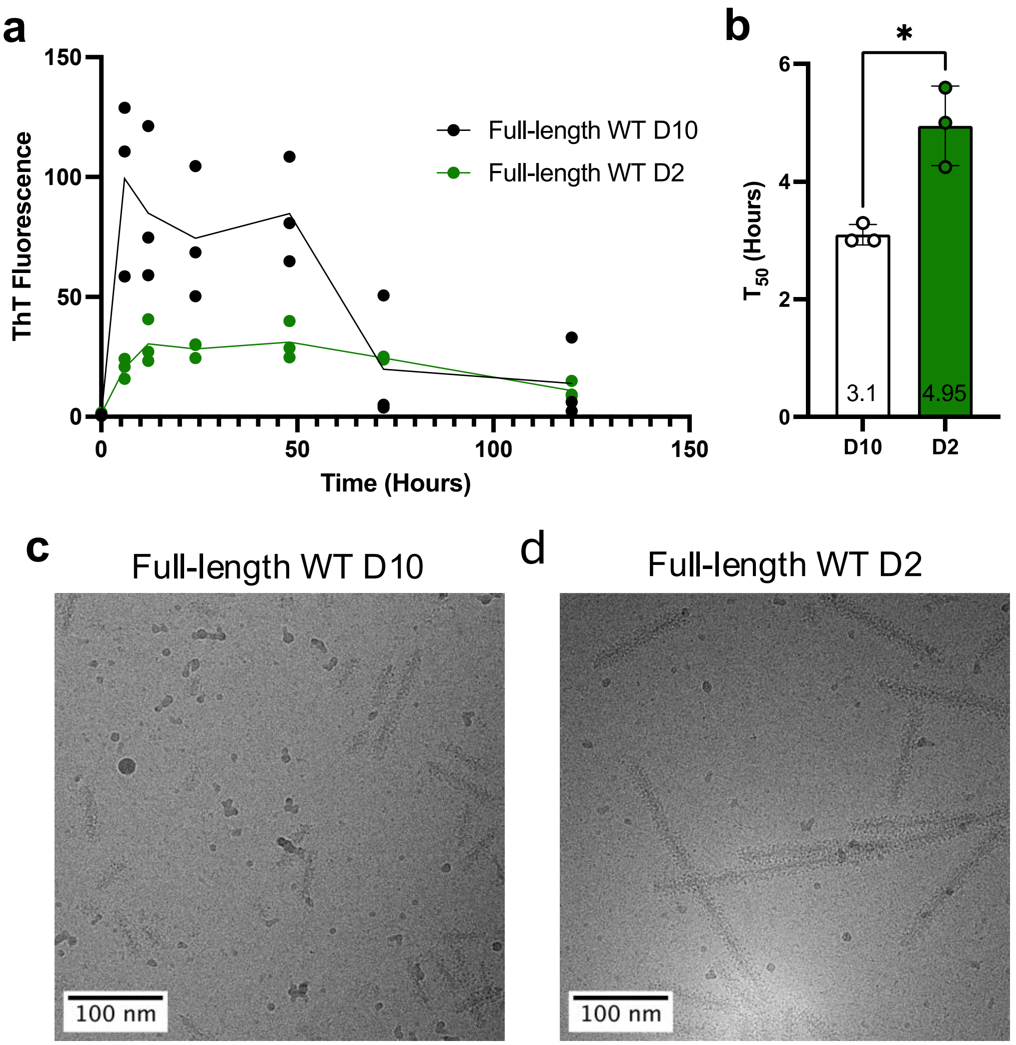

Supplementary Figure 1: Full length D10 and D2 fibrils. a) ThT monitored aggregation of purified recombinant full-length WT D10 (black) and D2 (green). **b**) ThT T_{50} for full-length WT D10 and D2 assembly (mean ±SD, n=3 biological replicates, unpaired two-tailed parametric Student's t-test; * = $p < 0.05$). **c**) Cryo-EM micrograph of aggregated full-length D10. **d**) Cryo-EM micrograph of aggregated full-length D2. Source data for **a** and **b** are provided as a Source Data file.

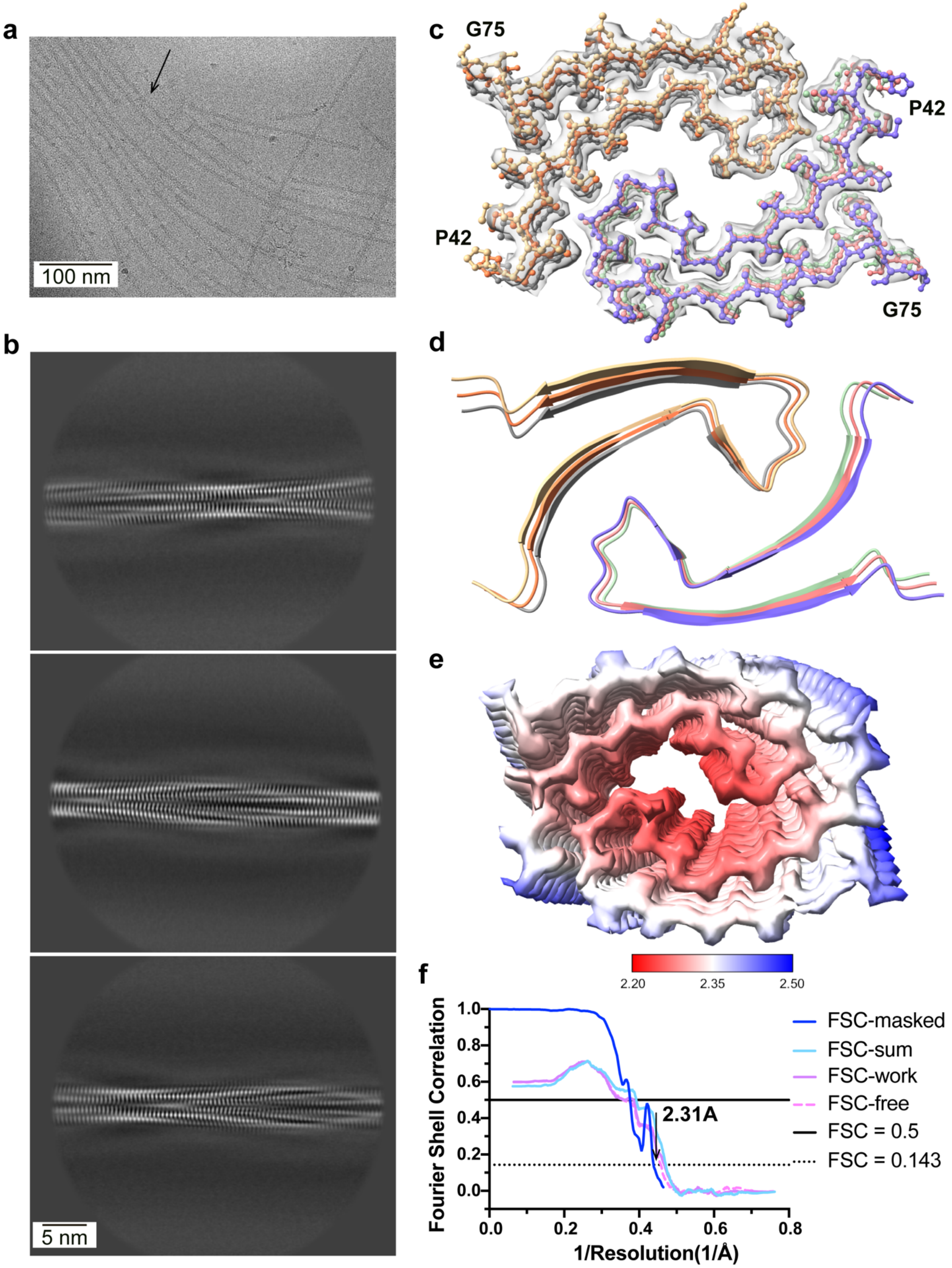

a
100 nm
b
5 nm
c
G75
P42
P42
G75
d
e
2.20
2.35
2.50
f
Fourier Shell Correlation
1/Resolution(1/Å)
2.31A
FSC-masked
FSC-sum
FSC-work
FSC-free
FSC = 0.5
FSC = 0.143

Supplementary Figure 2: Structure of D10-NT polymorph-1. a) Representative cryo-EM micrograph of D10-NT polymorph-1 fibrils. **b**) 2D class-averages used to generate initial model (box size 384 pixels, pixel size 1.076 Å) of D10-NT polymorph-1. **c**) Cryo-EM density map and atomic model of D10-NT polymorph-1. Three layers of the fibril structure are shown. **d**) Cartoon representation of secondary structure in the D10-NT polymorph-1 fibril core. **e**) Cryo-EM density map colored according to local resolution. **f**) Map and model validations, including FSC curves for the density map (blue), for the refined model versus full map (cyan), and for half maps for cross-validation (purple and pink). Black and dashed lines correspond to FSC values of 0.5 and 0.143, respectively. Source data for **f** are provided as a Source Data file.

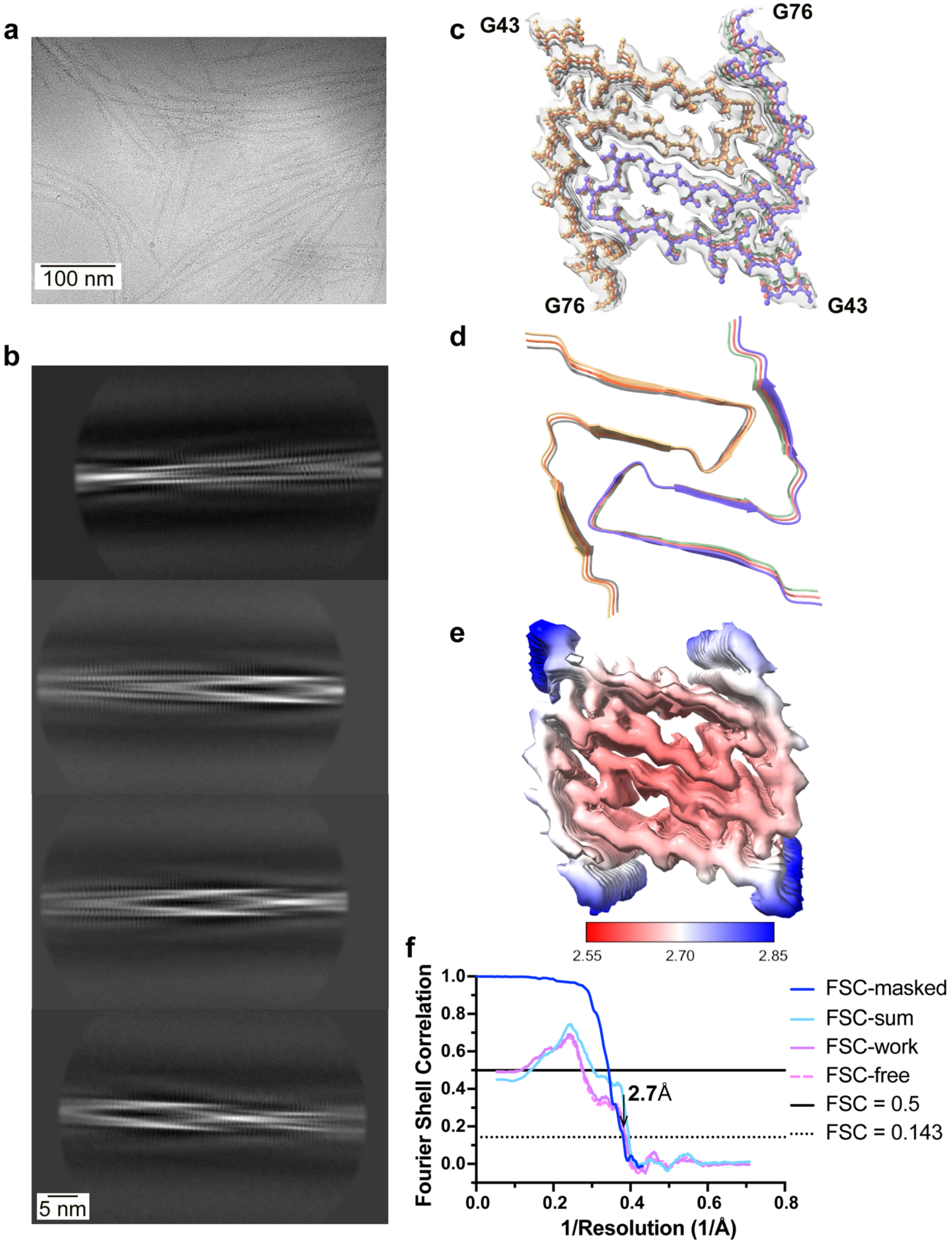

a
100 nm
b
5 nm
c
G43
G76
G76
G43
d
e
2.55
2.70
2.85
f
Fourier Shell Correlation
1/Resolution (1/Å)
2.7Å
FSC-masked
FSC-sum
FSC-work
FSC-free
FSC = 0.5
FSC = 0.143

Supplementary Figure 3: Structure of D10-NT polymorph-2. a) Representative cryo-EM micrograph of D10-NT polymorph-2 fibrils. **b**) 2D class-averages used to generate initial model (box size 512 pixels, pixel size 1.16 Å) of D10-NT polymorph-2. **c**) Cryo-EM density map and atomic model of D10-NT polymorph-2. Three layers of the fibril structure are shown. **d**) Cartoon representation of secondary structure in the D10-NT polymorph-2 fibril core. **e**) Cryo-EM density map colored according to local resolution. **f**) Map and model validations, including FSC curves for the density map (blue), for the refined model versus full map (cyan), and for half maps for cross-validation (purple and pink). Black and dashed lines correspond to FSC values of 0.5 and 0.143, respectively. Source data for **f** are provided as a Source Data file.

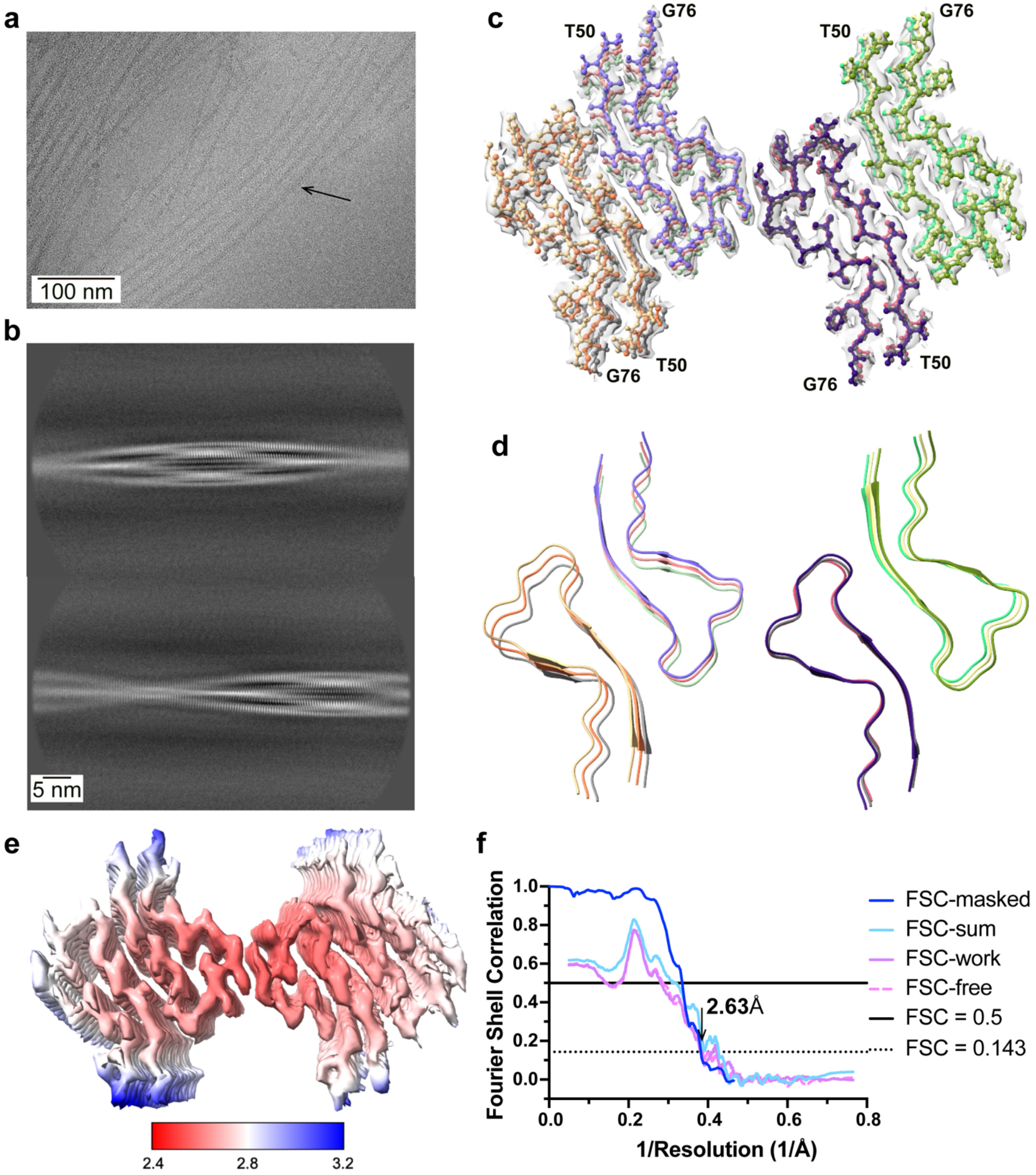

Supplementary Figure 4: Structure of D10-NT polymorph-3. **a**) Representative cryo-EM micrograph of D10-NT polymorph-3 fibrils. **b**) 2D class-averages used to generate initial model (box size 640 pixels, pixel size 1.076 Å) of D10-NT polymorph-3. **c**) Cryo-EM density map and atomic model of D10-NT polymorph-3. Three layers of the fibril structure are shown. **d**) Cartoon representation of secondary structure in the D10-NT polymorph-3 fibril core. **e**) Cryo-EM density map colored according to local resolution. **f**) Map and model validations, including FSC curves for the density map (blue), for the refined model versus full map (cyan), and for half maps for cross-

validation (purple and pink). Black and dashed lines correspond to FSC values of 0.5 and 0.143, respectively. Source data for **f** are provided as a Source Data file.

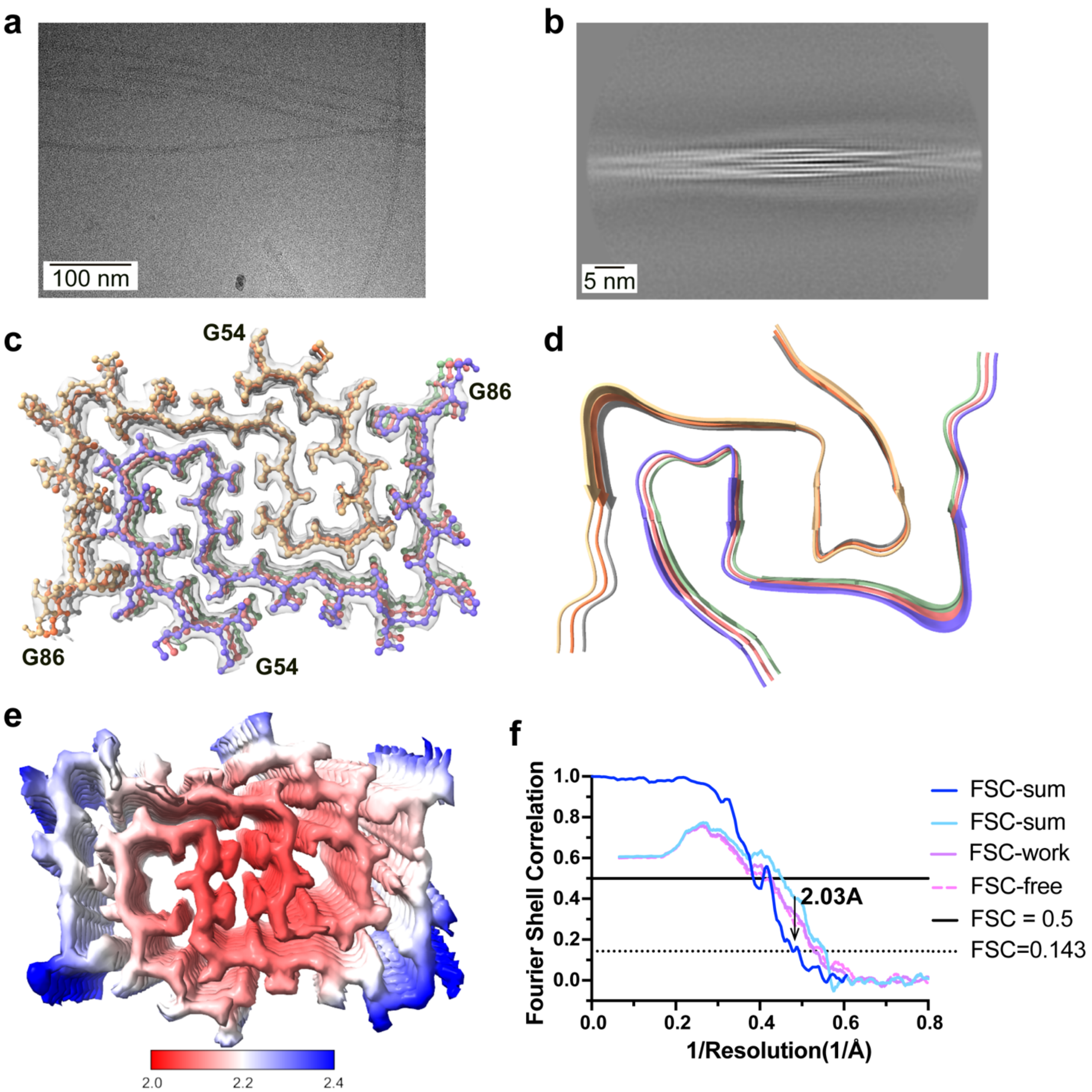

Supplementary Figure 5: Structure of D2-NT fibrils. a) Representative cryo-EM micrograph of D2-NT fibrils. **b**) 2D class-average used to generate initial model (box size 896 pixels, pixel size 0.825 Å) of D2-NT fibrils. **c**) Cryo-EM density map and atomic model of D2-NT fibrils. Three layers of the fibril structure are shown. **d**) Cartoon representation of secondary structure in the D2-NT fibril core. **e**) Cryo-EM density map colored according to local resolution. **f**) Map and model validations, including FSC curves for the density map (blue), for the refined model versus full map (cyan), and for half maps for cross-validation (purple and pink). Black and dashed lines correspond to FSC values of 0.5 and 0.143, respectively. Source data for **f** are provided as a Source Data file.

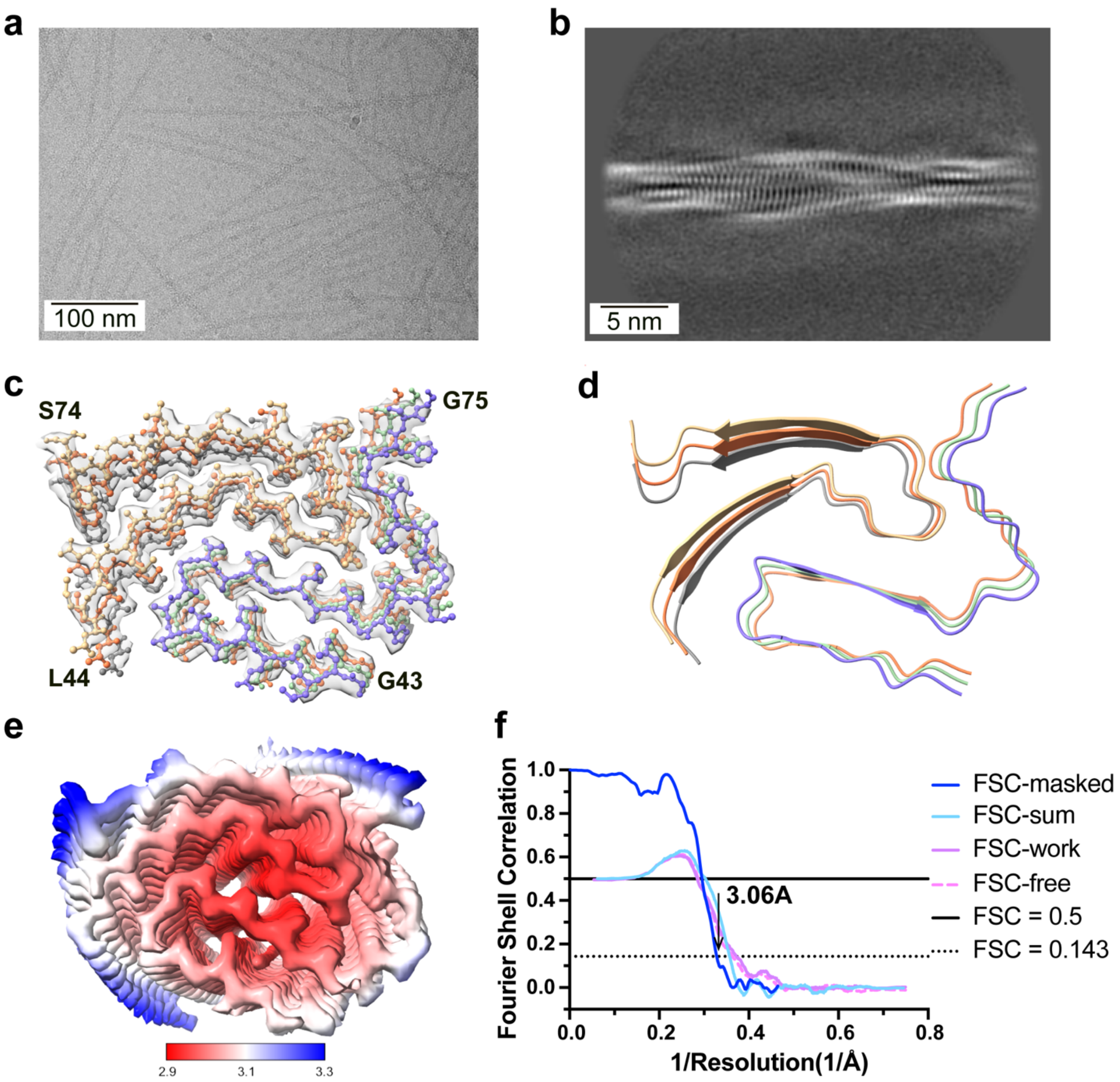

Supplementary Figure 6: Structure of S59L D10-NT fibrils. **a**) Representative cryo-EM micrograph of S59L D10-NT fibrils. **b**) 2D class-average used to generate initial model (box size 320 pixels, pixel size 1.076 Å) of S59L D10-NT fibrils. **c**) Cryo-EM density map and atomic model of S59L D10-NT fibrils. Three layers of the fibril structure are shown. **d**) Cartoon representation of secondary structure in the S59L D10-NT fibril core. **e**) Cryo-EM density map colored according to local resolution. **f**) Map and model validations, including FSC curves for the density map (blue), for the refined model versus full map (cyan), and for half maps for cross-validation (purple and pink). Black and dashed lines correspond to FSC values of 0.5 and 0.143, respectively. Source data for **f** are provided as a Source Data file.

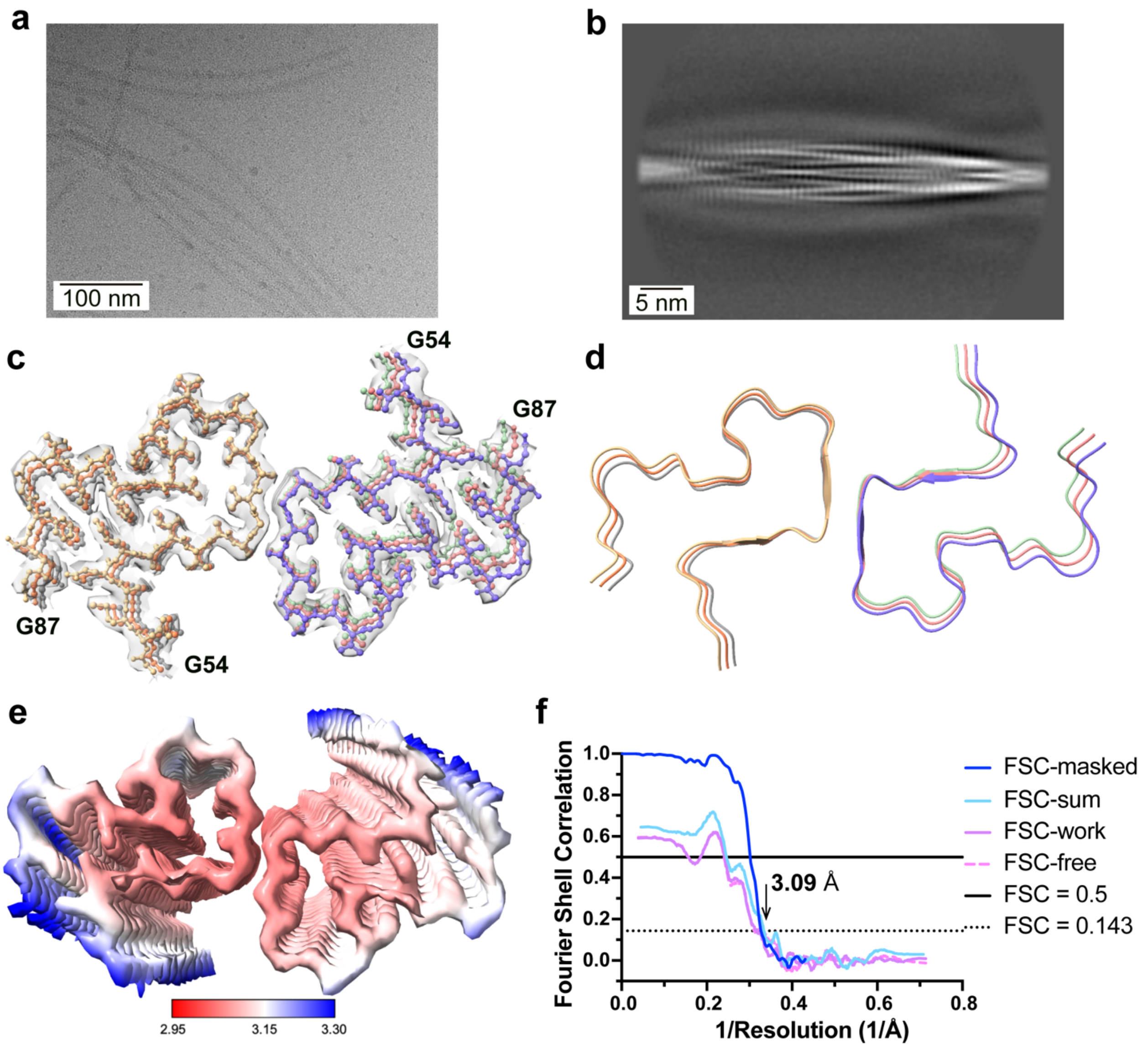

Supplementary Figure 7: Structure of T61I D2-NT fibrils. **a**) Representative cryo-EM micrograph of T61I D2-NT fibrils. **b**) 2D class-average used to generate initial model (box size 448 pixels, pixel size 1.16 Å) of T61I D2-NT fibrils. **c**) Cryo-EM density map and atomic model of T61I D2-NT fibrils. Three layers of the fibril structure are shown. **d**) Cartoon representation of secondary structure in the T61I D2-NT fibril core. **e**) Cryo-EM density map colored according to local resolution. **f**) Map and model validations, including FSC curves for the density map (blue), for the refined model versus full map (cyan), and for half maps for cross-validation (purple and pink). Black and dashed lines correspond to FSC values of 0.5 and 0.143, respectively. Source data for **f** are provided as a Source Data file.

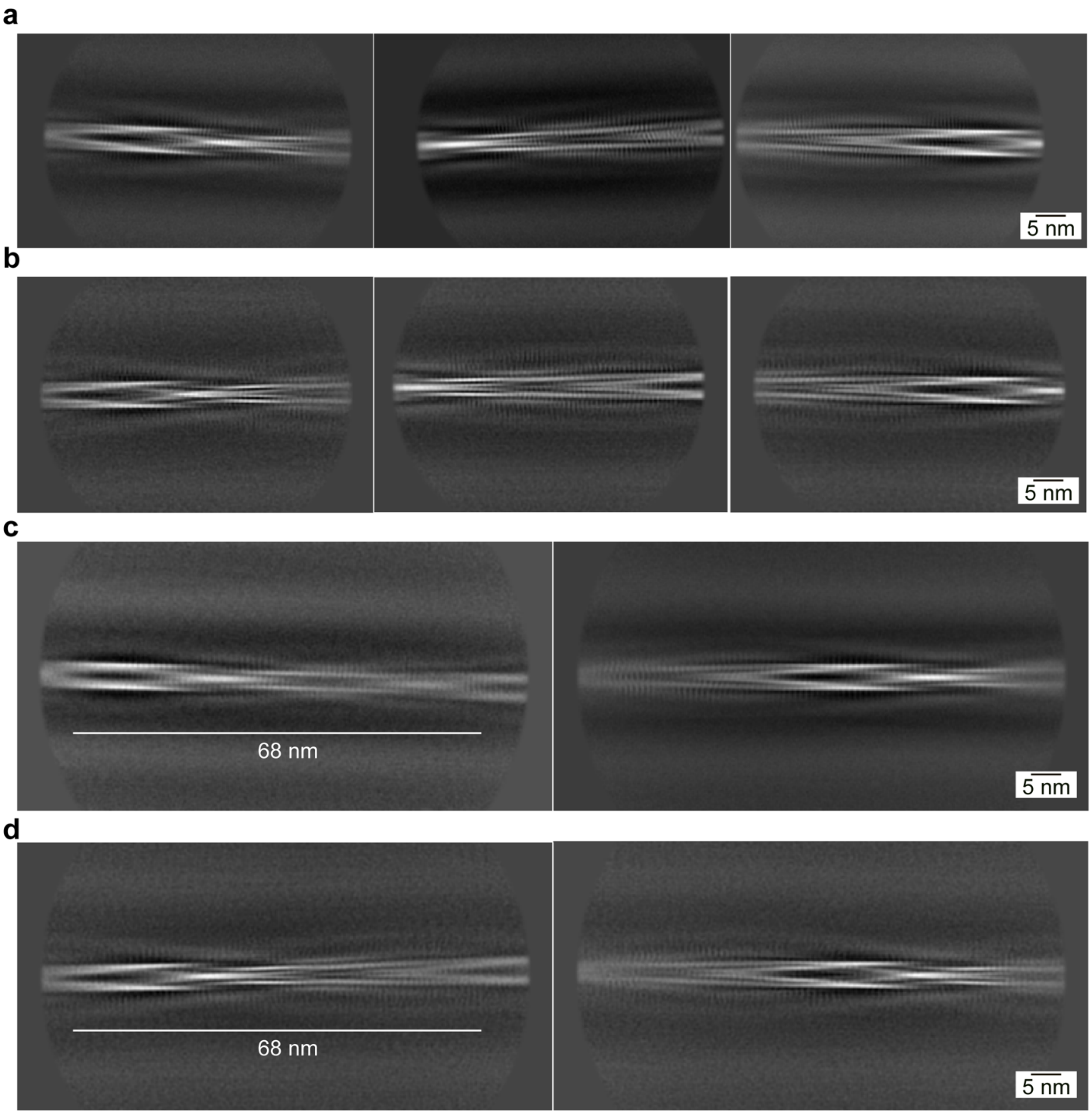

Supplementary Figure 8: Cyro-EM 2D classes of independent D10-NT sample. Comparison of 2D class averages from the WT D10-NT polymorph-2 structure dataset (**a**, **c**) with those from an independent WT D10-NT fibril sample dataset (**b**, **d**). The box size of the 2D classes is 512 pixels (pixel size 1.16 Å) in (**a**) and (**b**) and 768 pixels (pixel size 1.16 Å) in (**c**) and (**d**) is. The 180° cross-over distance estimated from the 2D classes using ImageJ is indicated.

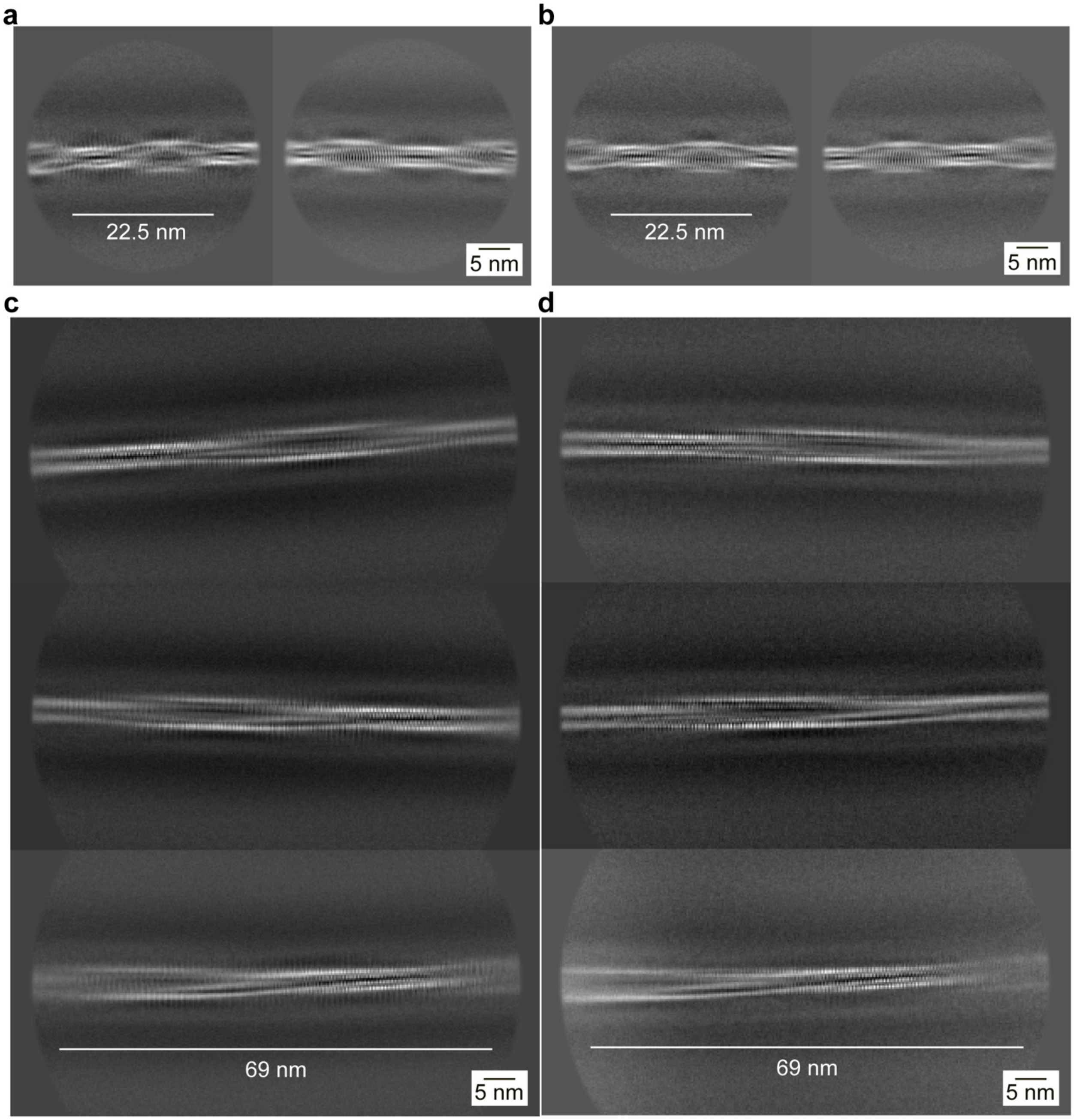

Supplementary Figure 9: Cyro-EM 2D classes of independent S59L D10-NT sample. Comparison of 2D class averages from conformation 1 (**a**, **c**) from the sample that resulted in the S59L structure with those (**b**, **d**) from an independent S59L D10-NT fibril sample dataset. The box size of the 2D classes is 384 pixels (pixel size 1.076 Å) in (**a**) and (**b**) and 784 pixels (pixel size 1.076 Å) in (**c**) and (**d**) is. The 180° cross-over distance estimated from the 2D classes using ImageJ is indicated.

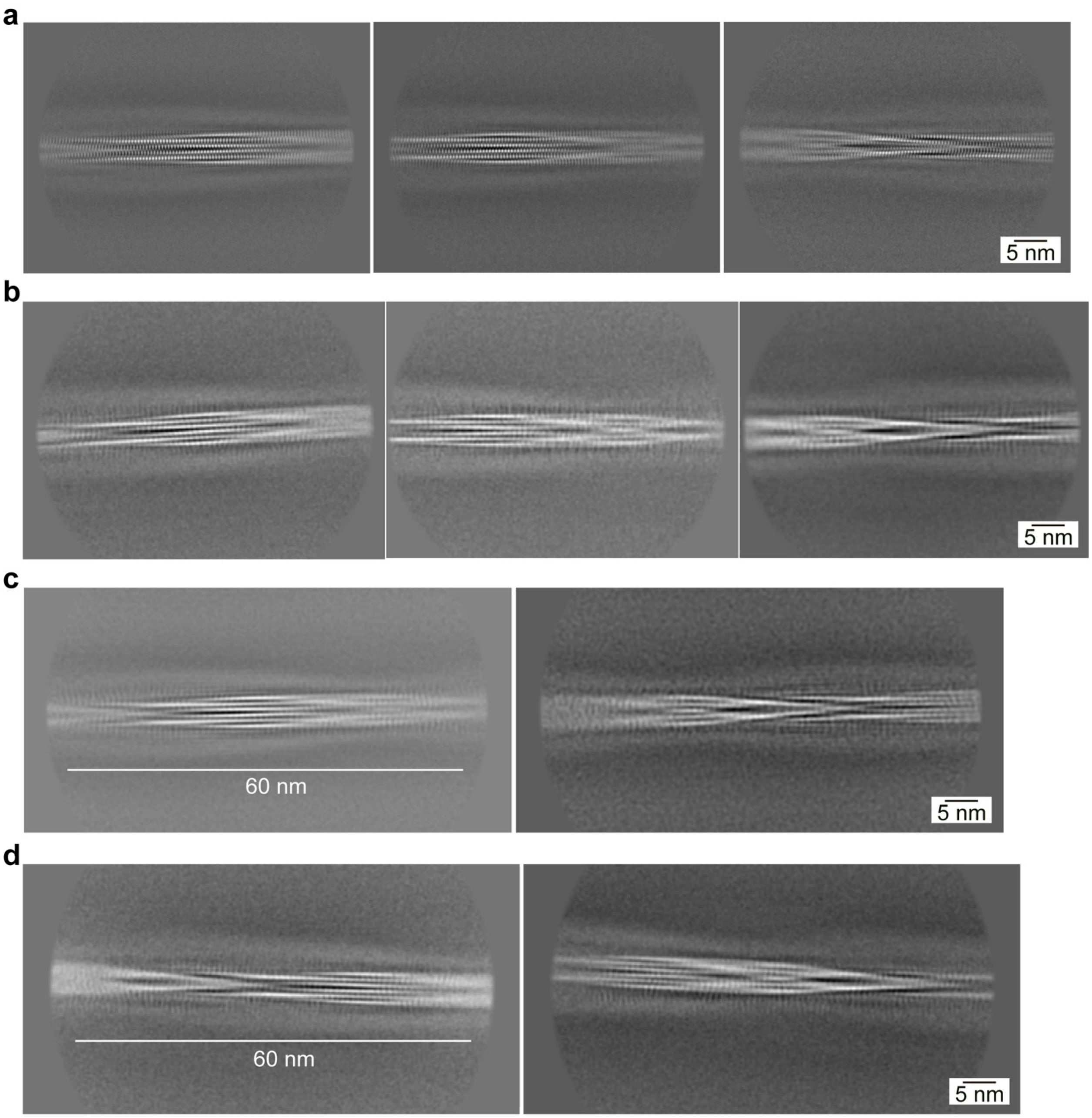

Supplementary Figure 10: Cyro-EM 2D classes of independent D2-NT sample. Comparison of 2D class averages from the WT D2-NT structure datast (**a, c**) with those from an independent WT D2-NT fibril sample dataset (**b, d**). The box size of the 2D classes is 640 pixels (pixel size 0.825 Å) in (**a**), 512 pixels (pixel size 1.076 Å) in (**b**), 896 pixels (pixel size 0.825 Å) in (**c**) and 700 pixels (pixel size 1.076 Å) in (**d**). The 180° cross-over distance estimated from the 2D classes using ImageJ is indicated.

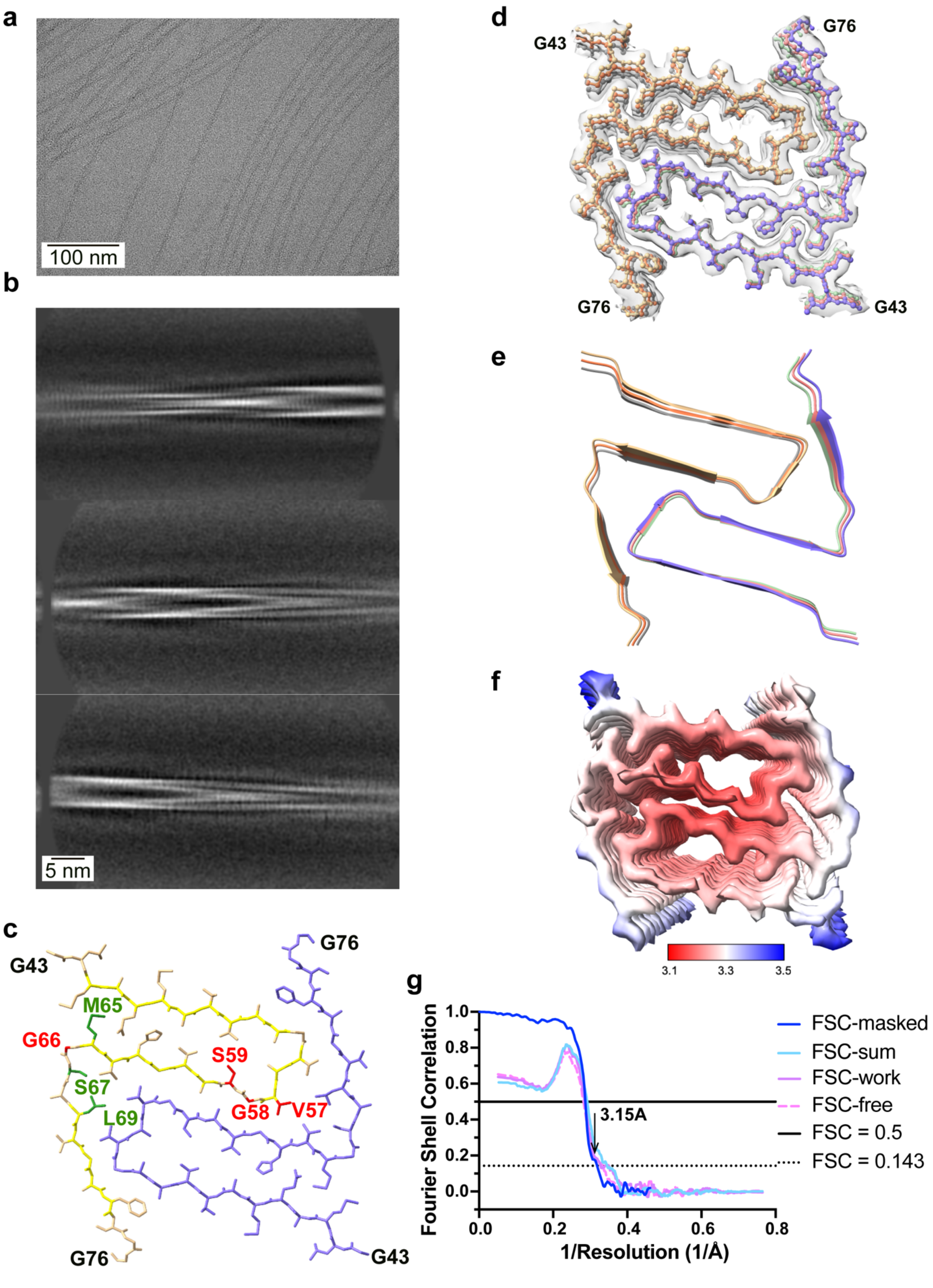
a
100 nm
b
5 nm
c
G43
G76
M65
G66
S59
S67
G58
V57
L69
G76
G43
d
G43
G76
G76
G43
e
f
3.1
3.3
3.5
g
Fourier Shell Correlation
1/Resolution (1/Å)
3.15A
FSC-masked
FSC-sum
FSC-work
FSC-free
FSC = 0.5
FSC = 0.143

Supplementary Figure 11: Structure of R15L D10-NT fibrils. **a**) Representative cryo-EM micrograph of R15L D10-NT fibrils. **b**) Representative 2D class-averages used to generate initial model (box size 512 pixels, pixel size 1.083 Å) of R15L D10-NT fibrils. **c**) Stick representation of the cryo-EM structure of a single layer of the ordered fibril core of R15L D10-NT fibrils. Beta strands are highlighted in yellow. Sites of disease linked mutations are shown in red. Amino acids that differ between D10 and D2 are shown in green. **d**) Cryo-EM density map and atomic model of R15L D10-NT fibrils. Three layers of the fibril structure are shown. **e**) Cartoon representation of secondary structure in the R15L D10-NT fibril core. **f**) Cryo-EM density map colored according to local resolution. **g**) Map and model validations, including FSC curves for the density map (blue), for the refined model versus full map (cyan), and for half maps for cross-validation (purple and pink). Black and dashed lines correspond to FSC values of 0.5 and 0.143, respectively. Source data for **g** are provided as a Source Data file.

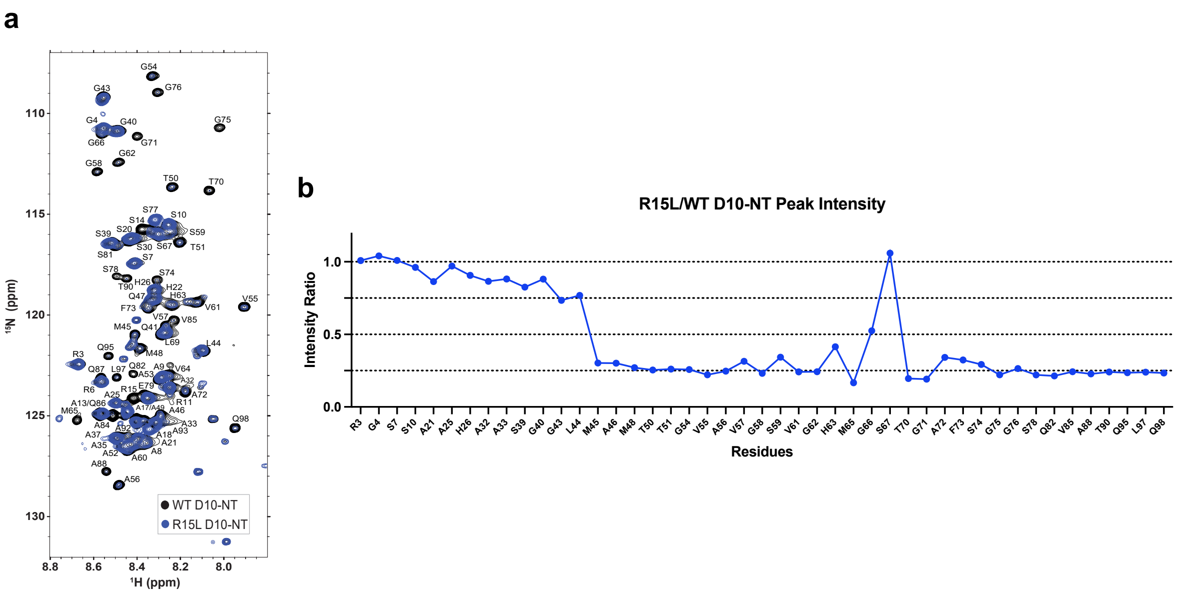

Supplementary Figure 12: NMR of R15L D10-NT. a) Overlayed NMR ^{15}N-^{1}H HSQC spectra of R15L (blue) and WT (black) D10-NT (see Supplementary Figure 19 for a full-page version). **b**) Ratios of R15L D10-NT NMR resonance intensities to those of corresponding WT D10-NT resonance intensities. Source data for **b** are provided as a Source Data file.

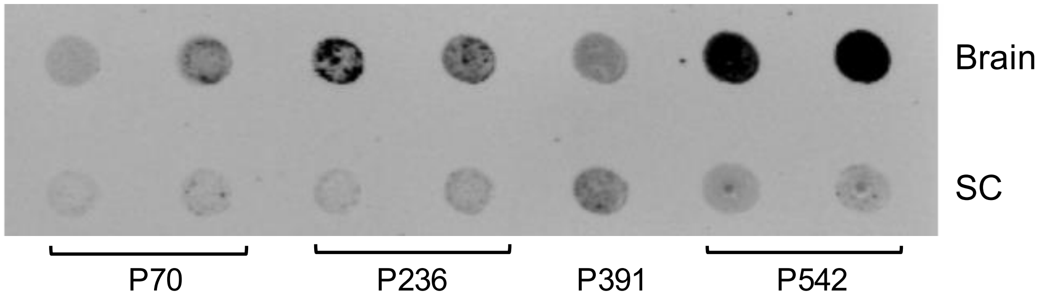

Supplementary Figure 13: D10 aggregates in very old WT mice. Filter trap capture of mitochondrial fractions from WT mouse brain and spinal cord (SC), at different ages (P70 (2 females), P236 (2 females), P391 (1 female) and P542 (2 male)) immunoblotted for D10. Aged mice have higher amounts of NP-40-insoluble material, especially in brain mitochondria. Results were obtained from a single experiment. See Supplementary Figure 20 for uncropped image.

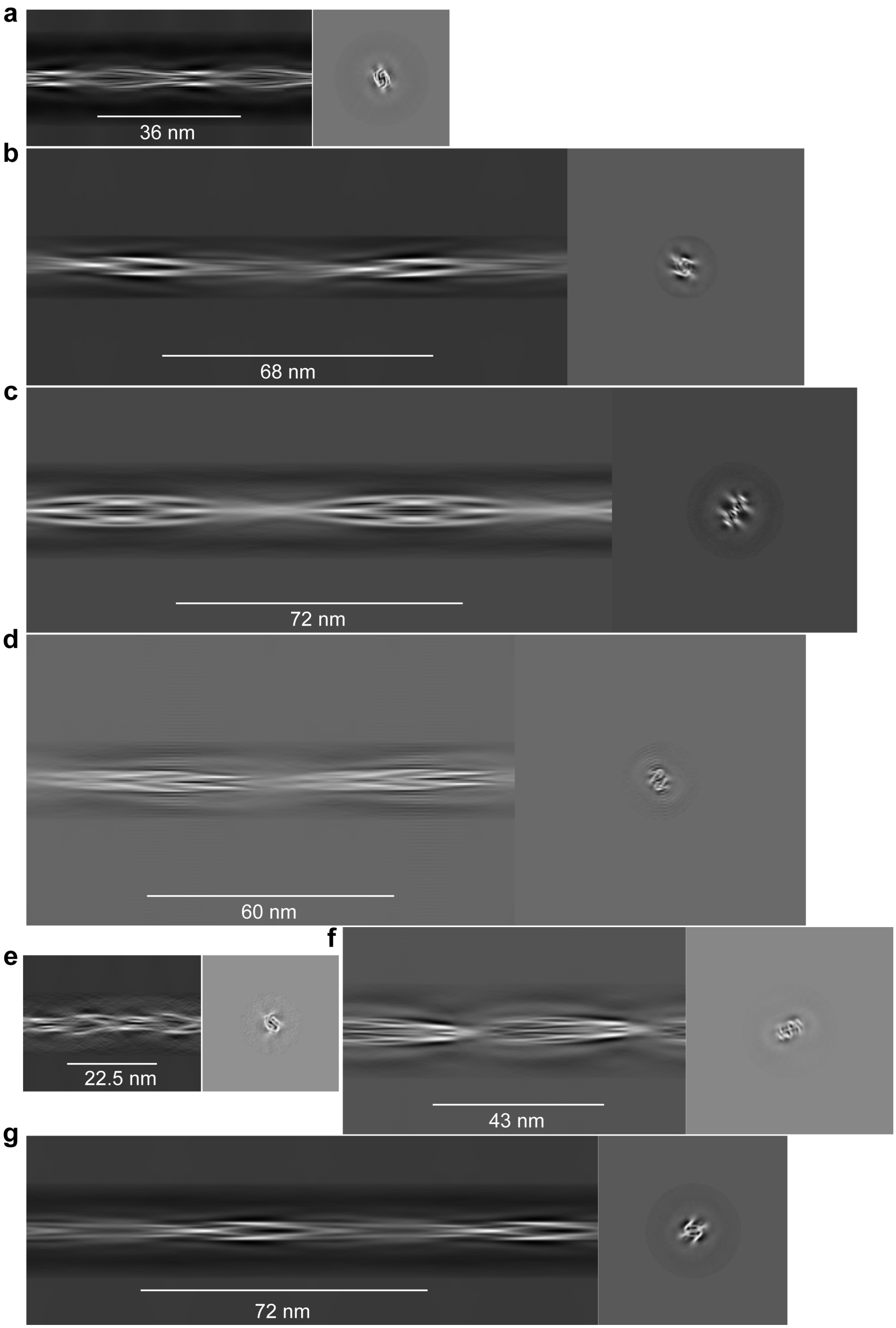
a
36 nm
b
68 nm
c
72 nm
d
60 nm
e
22.5 nm
f
43 nm
g
72 nm

Supplementary Figure 14: Initial models (2D projection and 2D reconstruction) for cryo-EM structures. WT D10-NT polymorph-1 (**a**), polymorph-2 (**b**), polymorph-3 (**c**), WT D2-NT (**d**), S59L D10-NT (**e**), T61I D2-NT (**f**), and R15L D10-NT (**g**). The 180°cross-over distance used to generate each initial model is indicated.

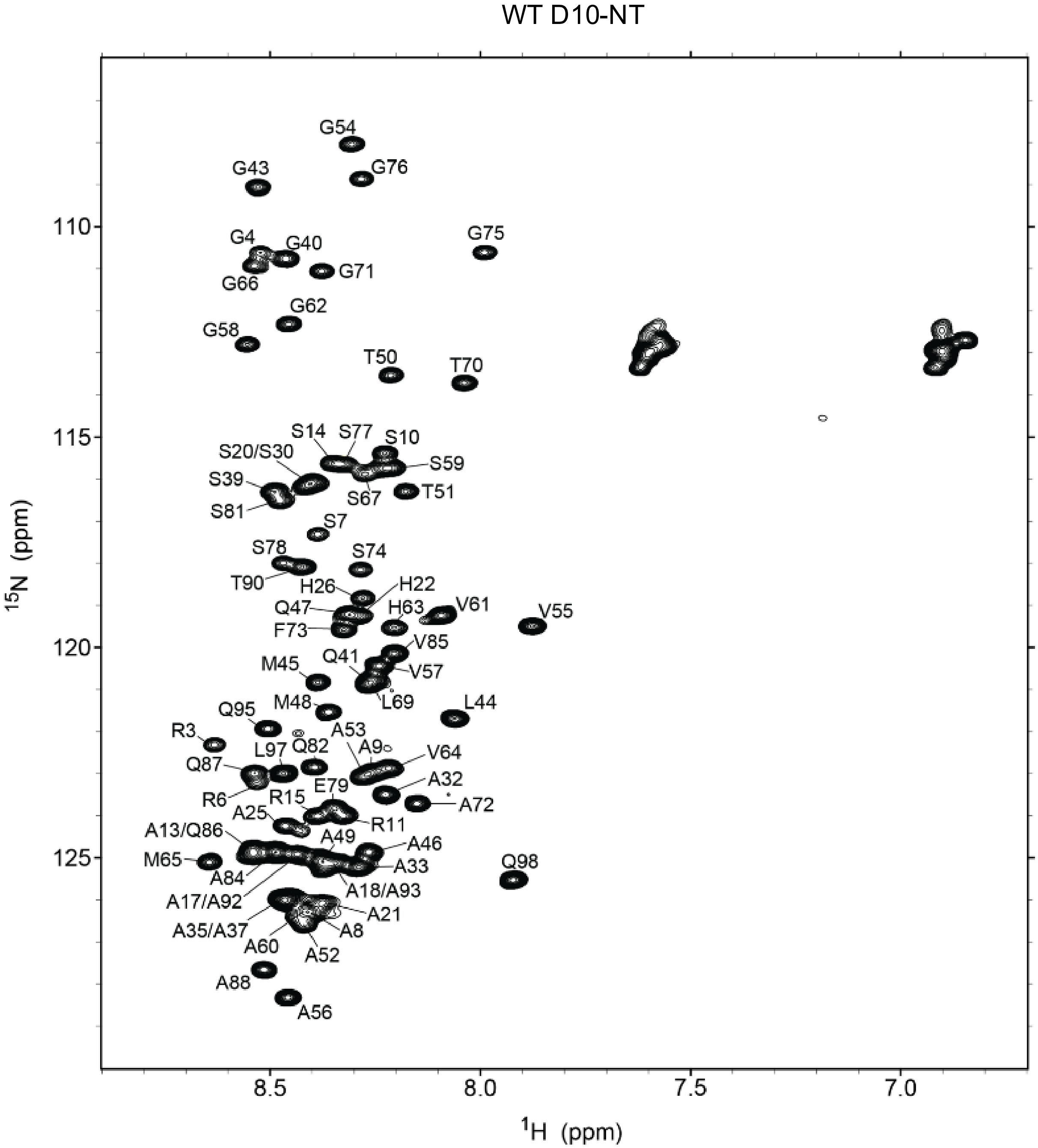

Supplementary Figure 15: NMR ^{15}N-^{1}H HSQC spectra of WT D10-NT. Full page version of Figure 1c.

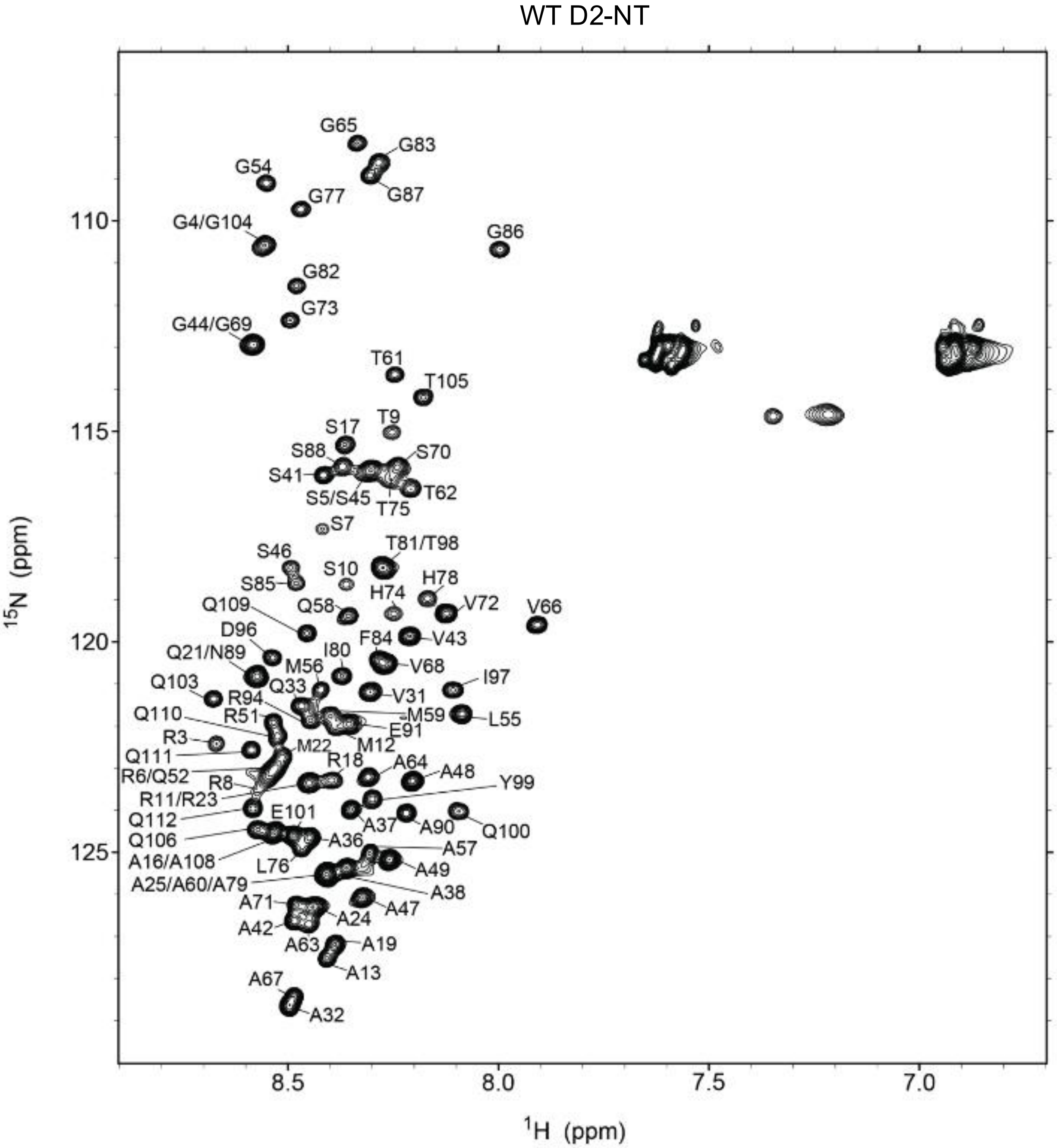

Supplementary Figure 16: NMR ^{15}N-^{1}H HSQC spectra of WT D2-NT. Full page version of Figure 1d.

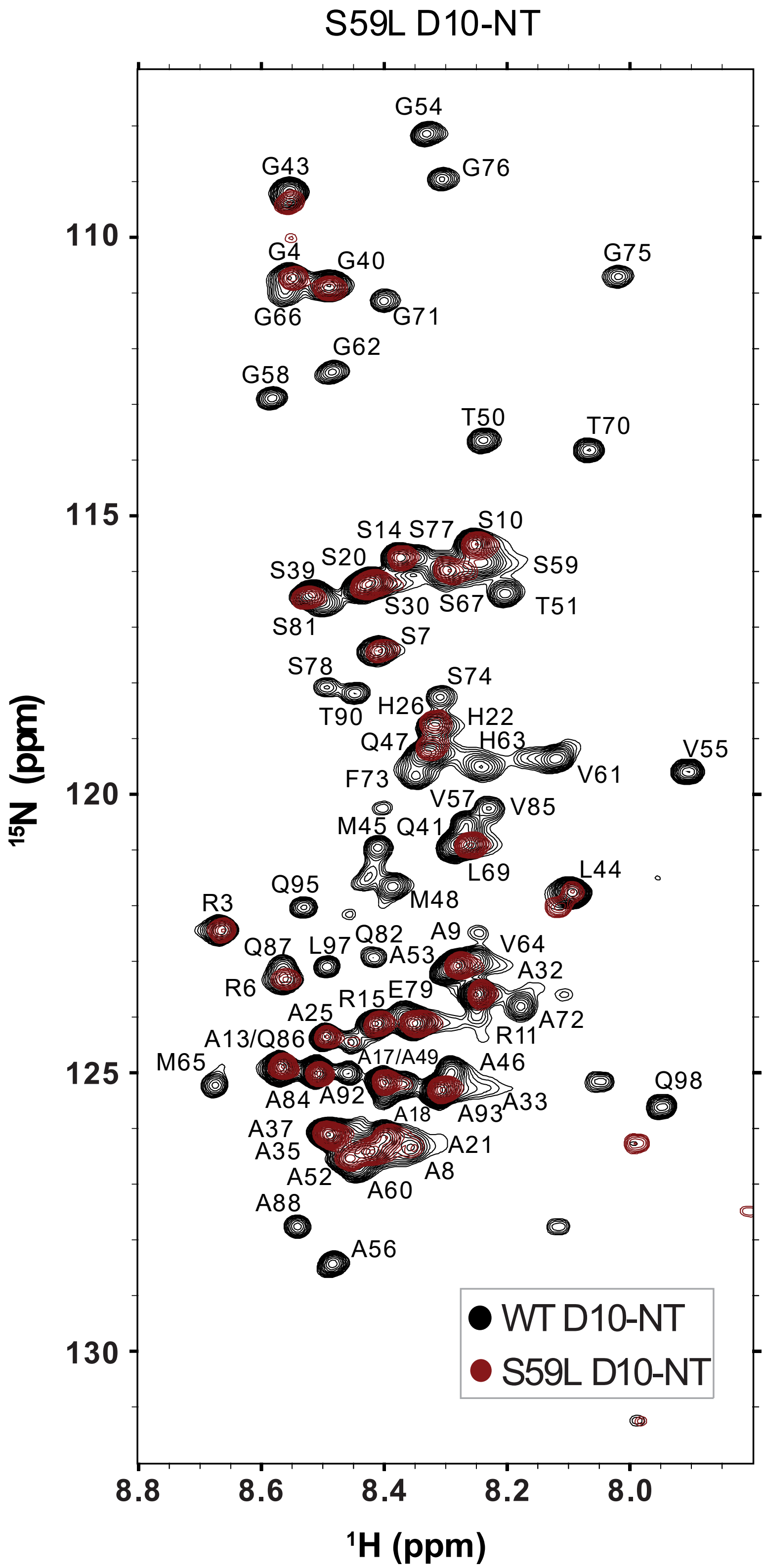

Supplementary Figure 17: Overlayed NMR ^{15}N-^{1}H HSQC spectra of S59L (red) and WT (black) D10-NT. Full page version of Figure 6e.

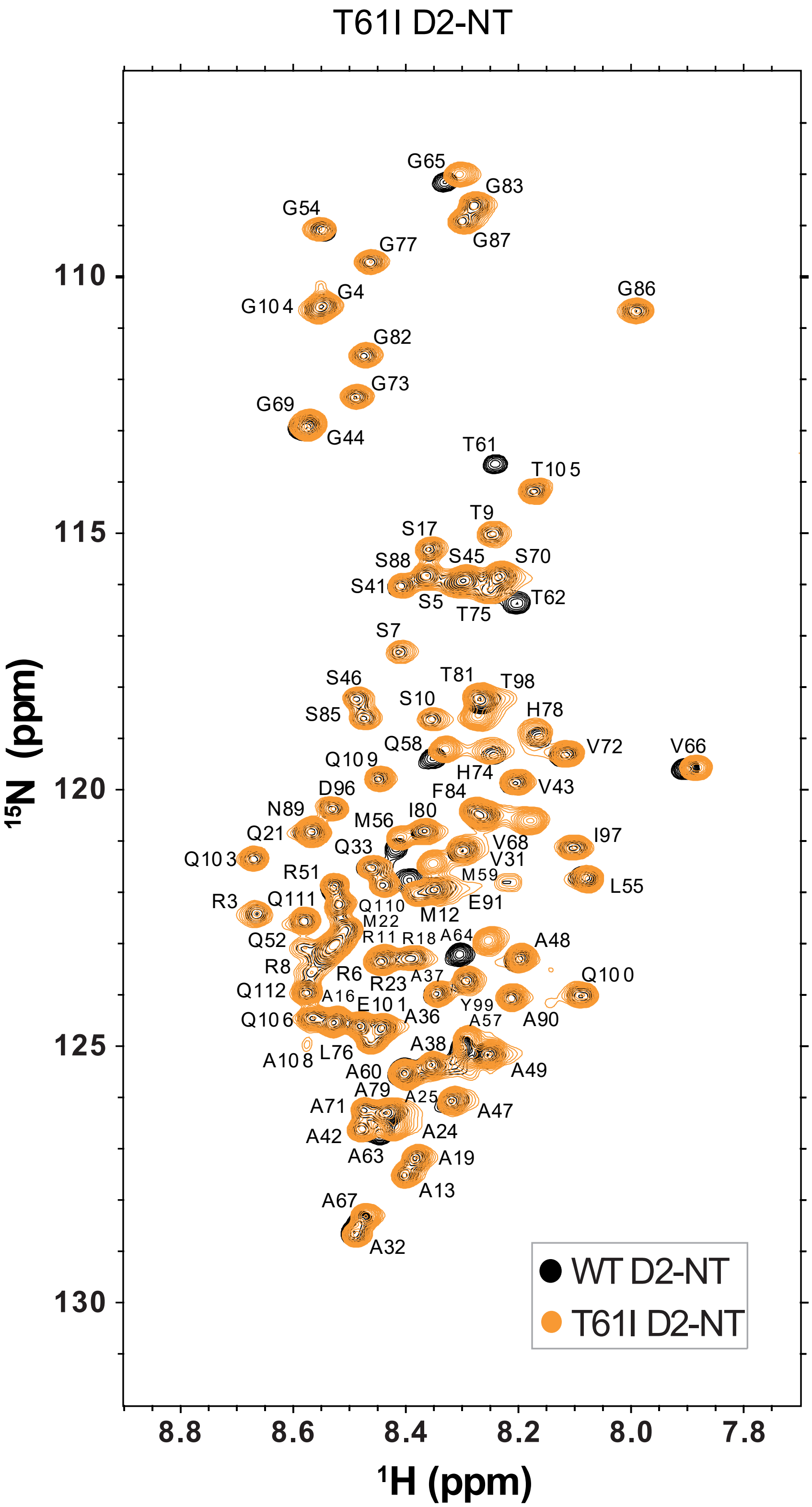

Supplementary Figure 18: Overlayed NMR ^{15}N-^{1}H HSQC spectra of T61I (orange) and WT (black) D2-NT. Full page version of Figure 6i.

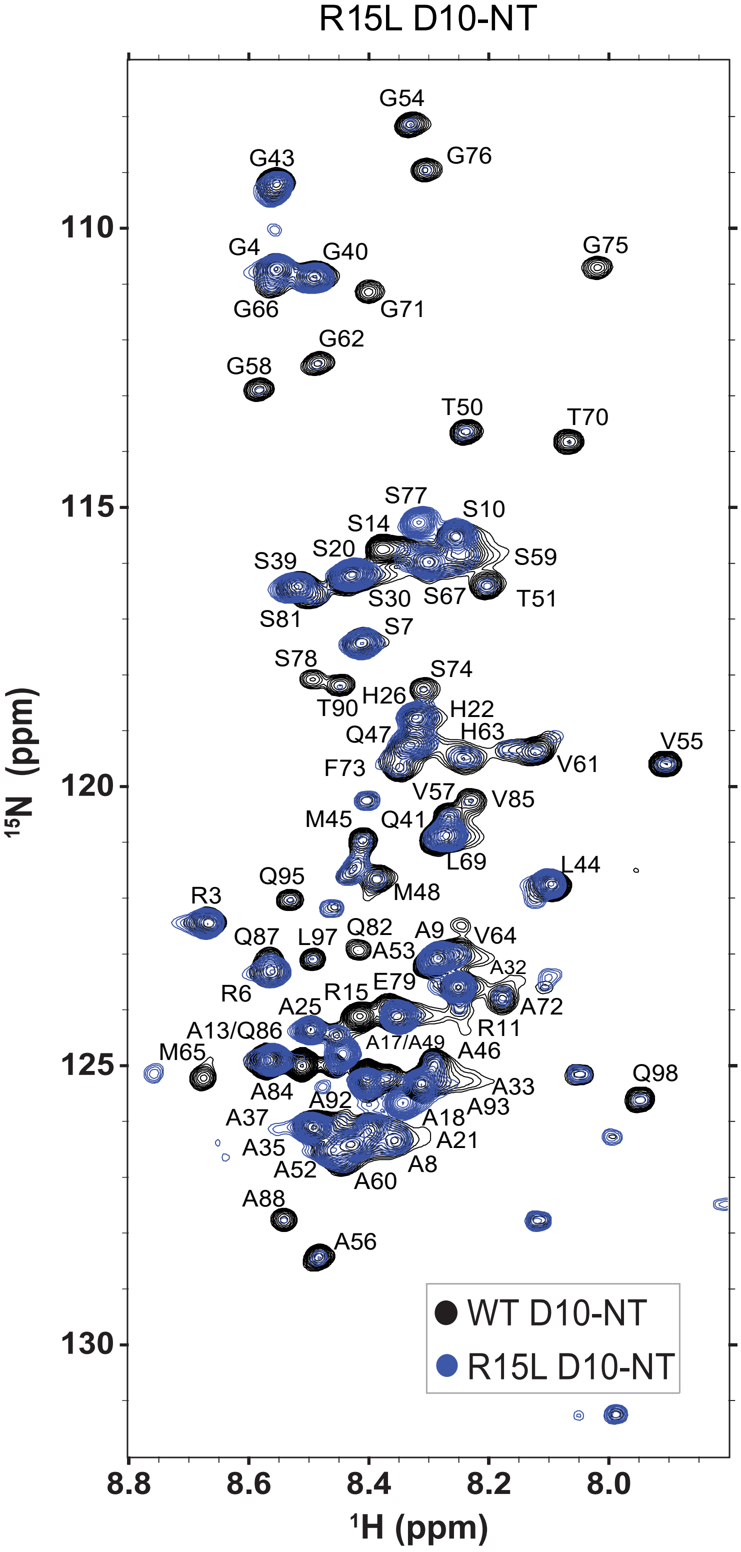

Supplementary Figure 19: Overlayed NMR ^{15}N-^{1}H HSQC spectra of R15L (blue) and WT (black) D10-NT. Full page version of Supplementary Figure 12a.

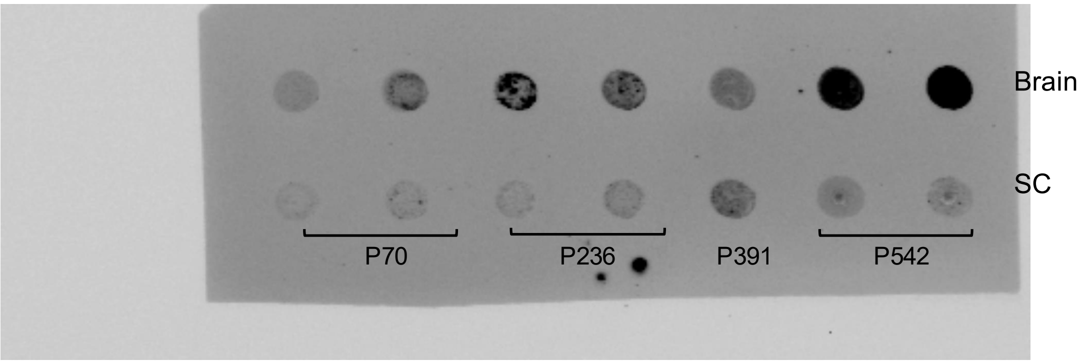

Supplementary Figure 20: Filter trap capture of mitochondrial fractions from WT mouse brain and spinal cord (SC), at different ages. Uncropped image of Supplementary Figure 13.

Supplementary Table 1: Summary of cryo-EM samples used to evaluate reproducibility

Sample	Concentration (μM)	Shaking speed (rpm)	Temp (°C)	Time point (hrs)	Polymorphs with twist*
D10-NT	80	1200	37	22	1
S59L D10-NT	80	1200	37	48	2
D2-NT	80	1200	37	48	1

*The number of polymorph classes was assessed using the FilamentTools procedure in RELION 5.0b.

Supplementary Table 2. Statistics of cryo-EM data collection for samples used to evaluate reproducibility

	D10-NT	S59L D10-NT	D2-NT
	**Data collection**		
Magnification	100,000	64,000	64,000
Pixel size (Å)	1.16	1.076	1.076
Defocus Range (μm)	0.8 to 2.5	0.8 to 2.5	0.8 to 3.0
Voltage (kV)	200	300	300
Energy filter	20 eV	20 eV	20 eV
Microscope/camera	Talos Glacios /Falcon 4i	Krios/K3	Krios/K3
Exposure time (s/frame)	0.1	0.05	0.05
Number of frames	48	44	40
Total dose ($e^-/Å^2$)	30.3	57.03	50
	**2D classification**		
Micrographs	1,800	5,935	6,071
Manually picked fibrils	333	749	3,440
Segments extracted (no.)	272,145	169,389	1,272,125
